# Curcumin Modifies the Activity of Plasmatic Antioxidant Enzymes and the Hippocampal Oxidative Profile in Rats upon Acute and Chronic Exposure to Ozone

**DOI:** 10.3390/molecules27144531

**Published:** 2022-07-15

**Authors:** Abraham Alberto Ramírez-Mendoza, Mario Alberto Ramírez-Herrera, Cesar Ricardo Cortez-Álvarez, Sendar Daniel Nery-Flores, Aldo Rafael Tejeda-Martínez, Marina María de Jesús Romero-Prado, María Luisa Mendoza-Magaña

**Affiliations:** 1 Laboratorio de Neurofisiología, Departamento de Fisiología, Centro Universitario de Ciencias de la Salud, Universidad de Guadalajara, Guadalajara 44340, Mexico; abrahamalberto.ramirez@alumnos.udg.mx (A.A.R.-M.); amario@cucs.udg.mx (M.A.R.-H.); marina.rprado@academicos.udg.mx (M.M.d.J.R.-P.); 2 Departamento de Farmacobiología, Centro Universitario de Ciencias Exactas e Ingenierías, Universidad de Guadalajara, Guadalajara 44430, Mexico; cesar.cortez@academicos.udg.mx; 3 Departamento de Investigación en Alimentos, Facultad de Ciencias Químicas, Universidad Autónoma de Coahuila, Saltillo 25280, Mexico; sendar.nery@uadec.edu.mx; 4 Laboratorio de Neurobiología Molecular, División de Neurociencias del Centro de Investigación Biomédica de Occidente, IMSS, Guadalajara 44340, Mexico; tejedamartinez.ar@gmail.com

**Keywords:** curcumin, antioxidant properties, ozone

## Abstract

Ozone (O_3_) is an oxidating tropospheric pollutant. When O_3_ interacts with biological substrates, reactive oxygen and nitrogen species (RONS) are formed. Severe oxidative damage exhausts the endogenous antioxidant system, which leads to the decreased activity of antioxidant enzymes such as catalase (CAT), glutathione peroxidase (GPx), and superoxide dismutase (SOD). Curcumin (CUR) is a natural polyphenol with well-documented antioxidant and anti-inflammatory properties. The aim of this work is to evaluate the effects of curcumin on CAT, GPx, and SOD activity and the inhibition of oxidative damage after the acute and chronic exposure to O_3_. Fifty male Wistar rats were divided into five experimental groups: the intact control, CUR-fed control, exposed-to-O_3_ control, CUR-fed (preventive), and CUR-fed (therapeutic) groups. These two last groups received a CUR-supplemented diet while exposed to O_3_. These experiments were performed during acute- and chronic-exposure phases. In the preventive and therapeutic groups, the activity of plasma CAT, GPx, and SOD was increased during both exposure phases, with slight differences; concomitantly, lipid peroxidation and protein carbonylation were inhibited. For this reason, we propose that CUR could be used to enhance the activity of the antioxidant system and to diminish the oxidative damage caused by exposure to O_3_.

## 1. Introduction

Globally, air pollution affects millions of people to such an extent that it has been associated with chronic degenerative diseases [1]. Among air pollutants, O_3_ stands out due to its high oxidizing power. O_3_ is produced via the photochemical reactions of volatile organic compounds and nitrogen oxides (NOx) [2]. When O_3_ interacts with the biologic epithelia, reactive oxygen and nitrogen species (RONS) are quickly produced. However, when these oxidative species are generated in low concentrations, they are efficiently inactivated by endogenous antioxidant enzymes. Furthermore, during acute exposure to O_3_, the endogenous antioxidant system is able to revert the oxidative damage once the exposure has ended [3,4,5]. Conversely, if RONS reach high concentrations and the exposure period becomes chronic, the antioxidant system is easily overcome, and oxidative damage is disseminated in tissues and organs. During these events, chained oxidative reactions occur through a variety of oxidant metabolites that affect most biomolecules [5,6]. Furthermore, the expression and activity of pro-oxidant enzymes (cyclo-oxygenase 2, lipo-oxygenase 5, and inducible nitroxide synthase) are increased by RONS through the activation of the nuclear factor kappa-light-chain-enhancer of activated B cells (NF-κB) [7]. Previous work by our group has demonstrated that NF-κB overactivation increases the concentration of inflammatory cytokines [8]. Similarly, the overactivation of NF-κB increases the expression of Kelch-like ECH-associated protein 1 (KEAP1), which binds to the nuclear factor erythroid 2-related factor 2 (Nrf2), leading to its proteosomal degradation. This fact could have consequences in decreasing the expression and activity of endogenous antioxidant enzymes [9]. On the other hand, the activation of Nrf2 increases the expression of antioxidant enzymes [10,11]. Among antioxidant enzymes, catalase (CAT), superoxide dismutase (SOD), and glutathione peroxidase (GPx) have the capacity to reduce RONS activity by blocking or delaying oxidative damage and protecting cellular components, such as proteins, lipids, carbohydrates, and DNA [10,12]. Despite the oxidative harmful effects caused by O_3_, there are few pharmacological approaches used to revert the damage process. The effects of several natural and synthetic molecules with antioxidant properties that might reduce the oxidative damage caused by RONS have been documented in the literature [13]. However, there are serious concerns regarding the secondary effects and adverse interactions that arise during their long-term consumption. For example, when vitamin C is administrated in the long term, it causes gastric disorders and ulcers. Vitamin E causes hemorrhagic events and mortality during long-term administration. Chronic melatonin administration has been related to daytime sleepiness, headaches, dizziness, and hypothermia [14,15,16]. Particularly, the effect of tibolone against the oxidative damage caused by ozone in the CNS has been evaluated and has demonstrated beneficial effects; however, the long-term administration of tibolone should be avoided due to an increased risk of different kind of cancers [17,18].

Curcumin (CUR) is a phenolic molecule extracted from *Curcuma longa* Lin rhizome, a plant that belongs to the Zingiberaceae family [19]. CUR exhibits potent antioxidant activity against RONS [20], and it can be administered for long periods of time without exerting side effects [21]. A variety of beneficial pharmacological effects of CUR, such as anticancer, antiparasitic, anti-inflammatory, and signaling pathway modulation effects, among others. These effects have been explained on the basis of multiple molecular targets on which CUR acts [19,22,23,24,25]. Thus, we are interested in studying this molecule and its activity in different damage models. CUR has been reported to increase the activity of antioxidant enzymes and to provide protection from oxidative damage [26]; however, this effect has not been studied in persistent oxidative damage during acute or chronic exposure to O_3_. In previously published articles, we demonstrated that curcumin (CUR) decreased NF-κB activation and reduced astrocytosis, microgliosis, and apoptosis in the hippocampus during acute and chronic O_3_ exposure in preventive and therapeutic approaches [8,27]. In fact, the hippocampus is crucial for short-term memory and learning, being one of the most important structures of the central nervous system, but it is also the most sensitive to oxidative damage, as it is directly connected to the olfactory mucosa, which is the first access site for exogenous pollutants [28]. The aim of this work is to evaluate effects of curcumin on the activity of plasma antioxidant enzymes and the oxidative profile in the hippocampi of rats exposed to acute and chronic doses of O_3_.

## 2. Results

### 2.1. Curcumin Modified Plasma Antioxidant Enzymes Activities during Ozone Exposure

#### 2.1.1. CAT Activity

**Figure 1:**
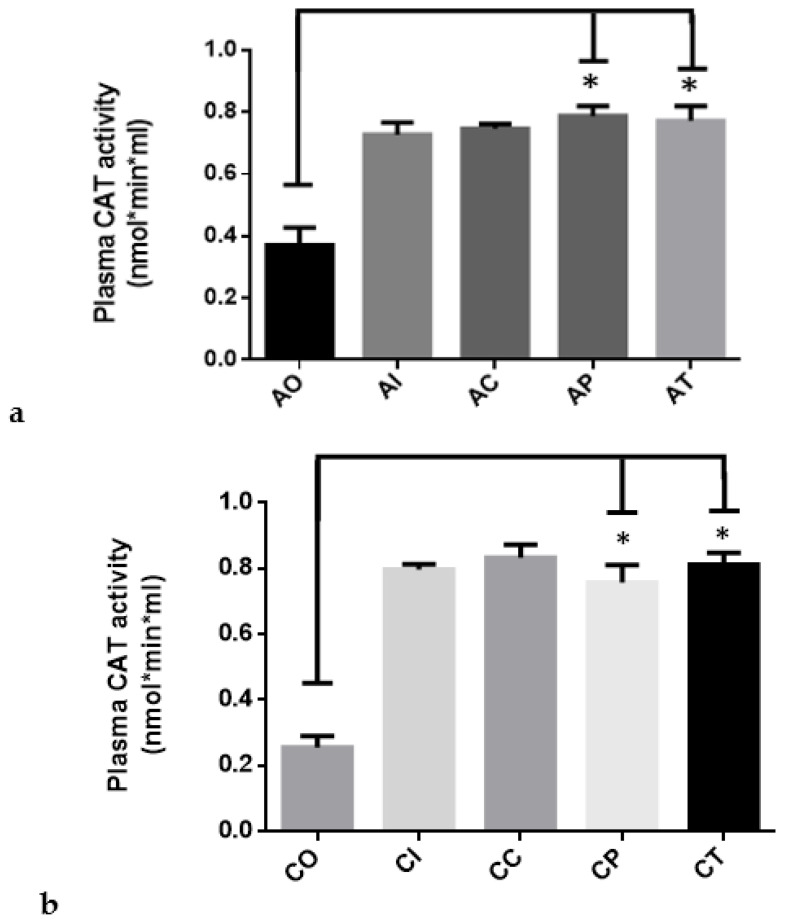
The catalase activity in rats exposed to ozone during the acute and chronic phases: (**a**) the preventive and therapeutic administration of dietary curcumin (5.6 mg/Kg) resulted in significantly increased activity (* *p* < 0.01) when evaluated at the end of the acute phase when compared to the control group exposed to ozone; acute O_3_ (AO), acute intact (AI), acute CUR (AC), acute preventive (AP), acute therapeutic (AT); (**b**) the effect of curcumin, when evaluated at the end of the chronic phase, maintained the same profile as observed in the acute phase, with a significance of *p* < 0.01 (*); chronic O_3_ (CO), chronic intact (CI), chronic CUR (CC), chronic preventive (CP), chronic therapeutic (CT).

CAT activity was determined by means of a spectrophotometric method that detects the presence of H_2_O_2_ in biological samples. Thus, a higher level of H_2_O_2_ means lower CAT activity. In Figure 1a shows that the plasma of rats exposed to O_3_ in the acute phase (AO, 0.3702 ± 0.057) had the lowest activity, which was significant when compared to the acute-intact group (0.7270 ± 0.040). In contrast, the dietary administration of CUR significantly improved the CAT activity in the AP (0.787 ± 0.031) and AT (0.772 ± 0.047) groups (*p* < 0.01). When compared against the control groups, AI and AC, AP and AT showed similar levels of activity.

In the chronic phase (Figure 1b), the CAT activity was similar to the acute phase. Again, dietary CUR caused a significant increase in CAT activity in the CP (0.757 ± 0.053) and CT (0.8115 ± 0.035) groups (*p* < 0.01) when compared to CO (0.255 ± 0.034), as observed in Figure 1b. When these groups were compared to the unexposed CI and CC groups, no significant differences were found.

#### 2.1.2. SOD Activity

**Figure 2:**
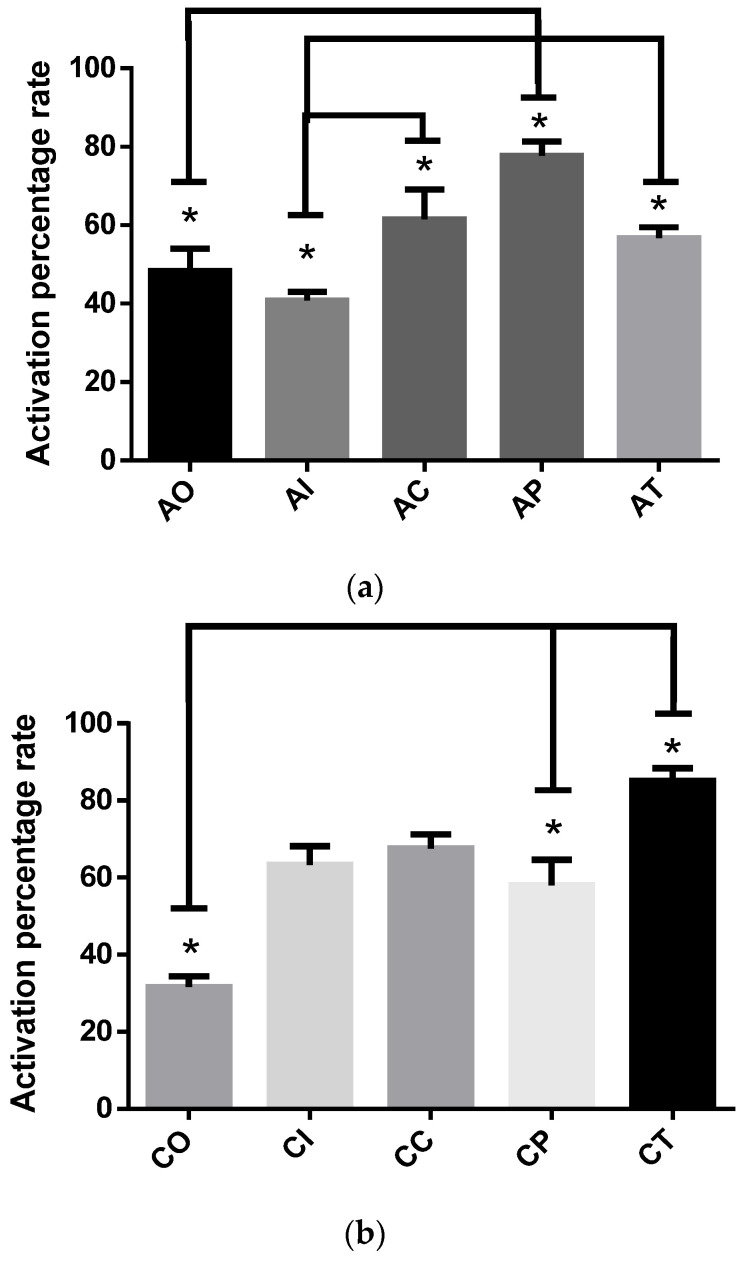
Activation percentage rate of superoxide dismutase in rats exposed to ozone during the acute and chronic phases: (**a**) The preventive dietary curcumin administration increased SOD activity (* *p* < 0.01) compared to AO, but the therapeutic administration failed to increase the SOD activity; acute O_3_ (AO), acute intact (AI), acute CUR (AC), acute preventive (AP), acute therapeutic (AT); (**b**) curcumin in CP and CT groups resulted in a significant SOD increase (* *p* < 0.01) compared to the CO group. The CI and CC groups exhibited significant increases in SOD activity versus the CO group (* *p* < 0.01); chronic O_3_ (CO), chronic intact (CI), chronic CUR (CC), chronic preventive (CP), chronic therapeutic (CT).

The SOD assay implies the use of the tetrazolium salt WST-1, which reacts with the superoxide anion generated by the xanthine oxidase. Thus, the dismutation of the superoxide anion into H_2_O_2_ and O_2_ catalyzed by SOD is related to WST-1 reducing into WST-1 formazan. The resulting data are expressed in terms of the activity percentage rate. Figure 2a depicts that the effects of dietary CUR on SOD activity significantly increased in the AP group (77.74 ± 3.62), as the percentage of the activation rate increased significantly (*p* < 0.01) versus the OA group (48.34 ± 5.72). The AI group (40.83 ± 2.20, *p* < 0.01) also showed lower SOD activity compared to the AC group; thus, the increased SOD activity in the AC group could be due to a CUR effect. Additionally, the effect of dietary CUR in the AT group also increased SOD activity versus the AI group (*p* < 0.01).

After the chronic phase was completed (Figure 2b), dietary CUR significantly increased SOD activity in the CP (58.02 ± 6.64) and CT (85.0 ± 3.41) groups (*p* < 0.01) when compared to the CO group (31.67 ± 2.68). Furthermore, the CT group showed better SOD activity when compared to CP (*p* < 0.05). The groups CI (63.33 ± 4.83) and CC (67.50 ± 3.70) also showed increased SOD activity (*p* < 0.01) compared to the group exposed to O_3_ (CO). Additionally, the SOD activity was significantly increased in the CI group (panel b) when versus the AI group (panel a).

#### 2.1.3. GPx Activity

**Figure 3:**
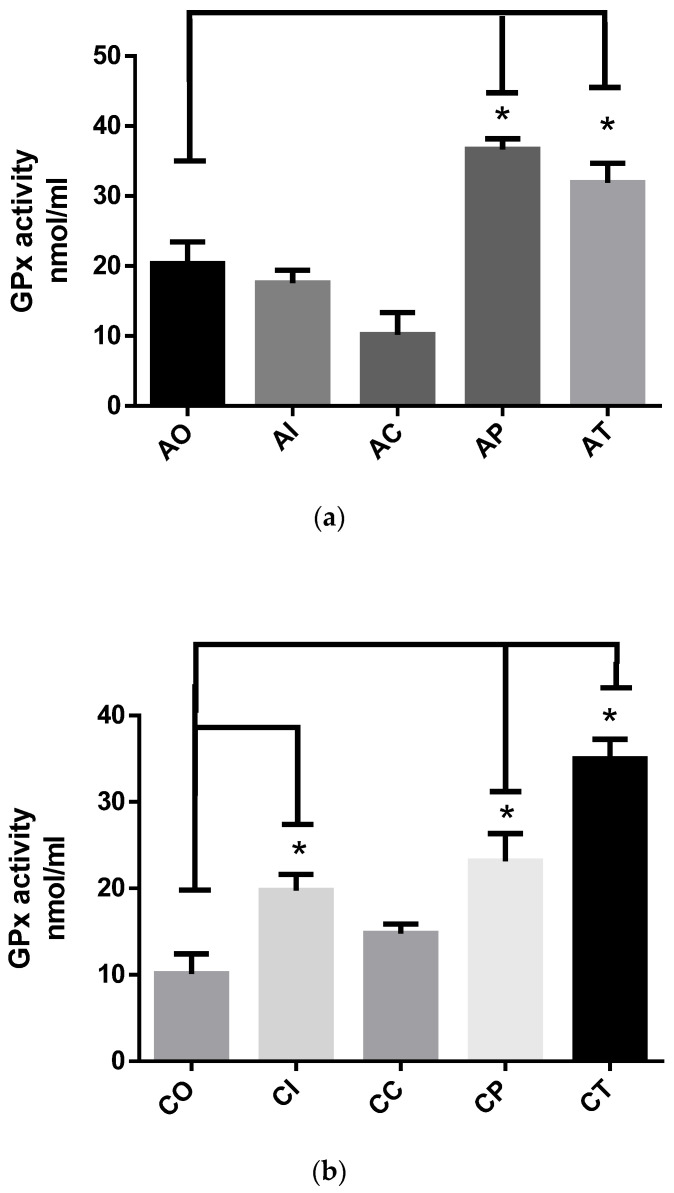
The glutathione peroxidase (GPx) activity in rats exposed to ozone during the acute and chronic phases: (**a**) the preventive administration of dietary curcumin in the preventive approach at the end of the acute phase presented increased activity of GPx (* *p* < 0.05) when compared to the AO group. Similarly, the AT group showed increased GPx activity (*p* < 0.01) compared to the acute ozone-exposed control group OA; acute O_3_ (AO), acute intact (AI), acute CUR (AC), acute preventive (AP), acute therapeutic (AT); (**b**) the effect of curcumin in the CP and CT groups (* *p* < 0.01) resulted significantly increased GPx activity (* *p* < 0.05) compared to the chronic ozone-exposed control CO group; chronic O_3_ (CO), chronic intact (CI), chronic CUR (CC), chronic preventive (CP), chronic therapeutic (CT).

The GPx assay is mainly based on the oxidation of GSH to generate GSSG, inducing the reduction of cumene hydroperoxide. The glutathione reductase reduces GSSG to produce GSH, thereby consuming NADPH. The decrease in NADPH is proportional to the GPx activity. In the acute phase (Figure 3a), dietary CUR significantly increased the GPx activity in the AP (36.63 ± 1.55, *p* < 0.01) and AT (31.90 ± 2.77, *p* < 0.05) groups compared to the AO group (20.37 ± 3.08). The AI and AC groups showed low GPx activity, similar to the AO group. In the chronic phase (Figure 3b), the CP group (23.14 ± 3.21) and CT group (34.97 ± 2.27) exhibited a significant increase in GPx activity caused by CUR (*p* < 0.01) compared to the CO group (10.11 ± 2.31). The CI group (19.72 ± 1.88) also showed a significant increase in GPx activity when compared to the CO group (*p* < 0.01).

#### 2.1.4. Inhibition of MDA and 4-HNE Formation by CUR

**Figure 4:**
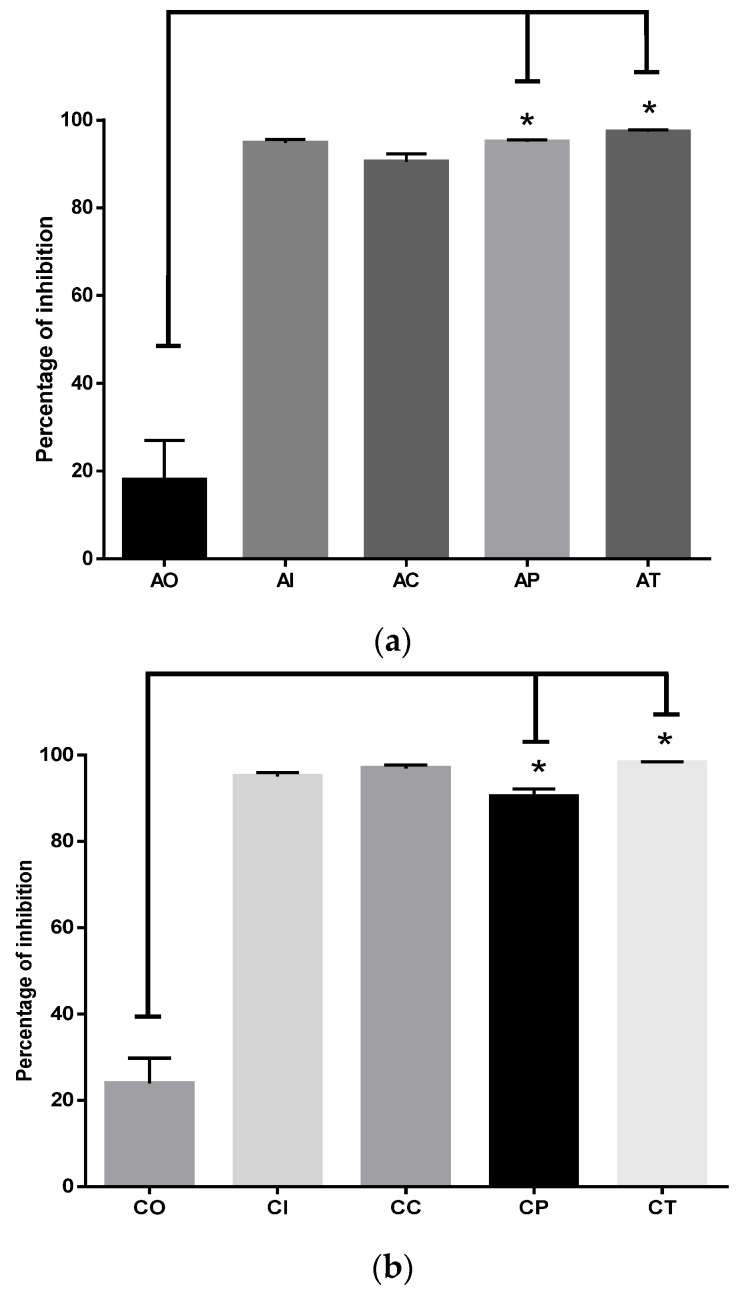
Inhibition of oxidative damage to lipids by curcumin in acute and chronic phases after exposure to ozone: (**a**) in the acute exposure to ozone, the administration of dietary curcumin in preventive and therapeutic modes significantly inhibited the oxidative damage to lipids (* *p* < 0.01) compared to the control group exposed to ozone OA; acute O_3_ (AO), acute intact (AI), acute CUR (AC), acute preventive (AP), acute therapeutic (AT); (**b**) the effect of curcumin in preventive and therapeutic administration significantly inhibited oxidative damage to lipids (* *p* < 0.01), compared to the CO group; chronic O_3_ (CO), chronic intact (CI), chronic CUR (CC), chronic preventive (CP), chronic therapeutic (CT).

The oxidative damage to hippocampal lipids was significantly inhibited by CUR in the acute phase (Figure 4a). Comparing the percentages in the AO group (18.07% ± 8.89) versus the percentages of the AP (95.02% ± 0.4638) and AT (97.35% ± 0.4279) groups, the difference was statistically significant (*p* < 0.0001). There were no significant differences among the AP and AT groups versus the AI and AC groups. In the chronic phase (Figure 4b), the percentage of oxidative damage to lipids depicted a similar profile, in which the CP group (90.40% ± 1.765) and the CT group (98.23% ± 0.222) showed significant inhibition caused by CUR (*p* < 0.0001) compared to CO (23.92% ± 5.847).

#### 2.1.5. Curcumin Inhibited Oxidative Damage to Hippocampal Proteins

**Figure 5:**
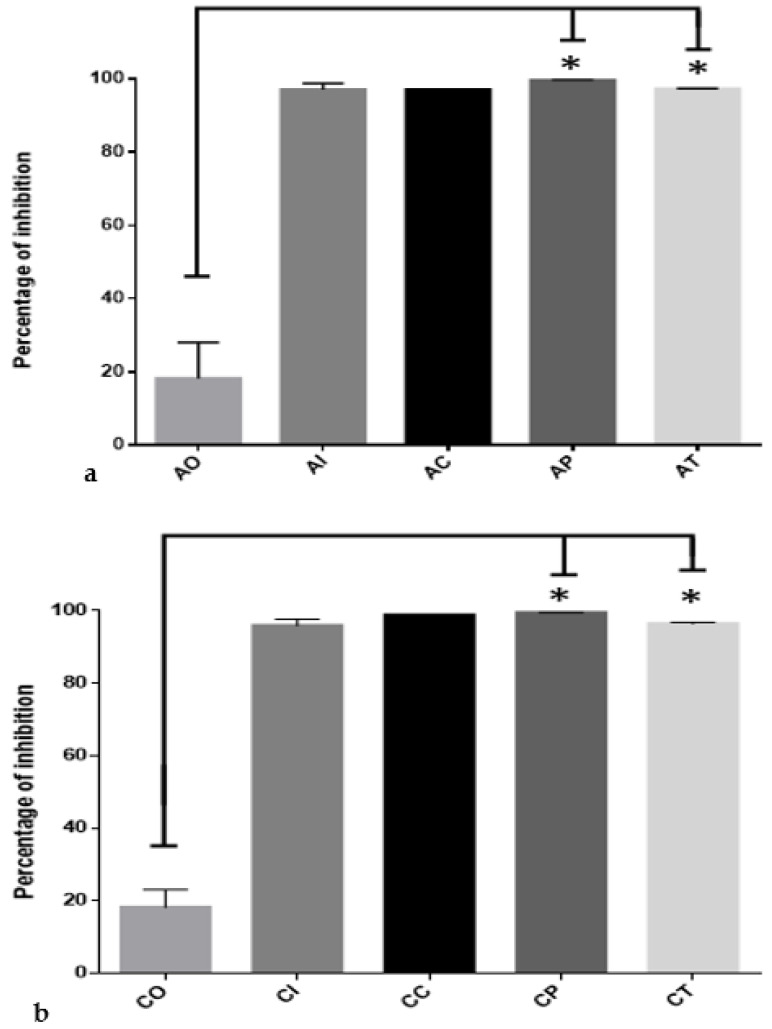
Inhibition of oxidative damage to hippocampal proteins exerted by curcumin against the ozone exposure in the acute and chronic phases: (**a**) the administration of dietary curcumin to the AP and AT groups significantly inhibited (* *p* < 0.0001) protein oxidation of hippocampal tissue in rats exposed to ozone when compared to the OA group, where oxidative damage was at the maximum level; acute O_3_ (AO), acute intact (AI), acute CUR (AC), acute preventive (AP), acute therapeutic (AT); (**b**) this image depicts the effect of curcumin in preventive and therapeutic administration during chronic exposure to ozone; in both cases, the oxidative damage was markedly inhibited (* *p* < 0.0001) compared to damage determined in the chronic ozone-exposure control group (CO); chronic O_3_ (CO), chronic intact (CI), chronic CUR (CC), chronic preventive (CP), chronic therapeutic (CT).

Dietary administration of CUR protected hippocampal proteins during exposure to O_3_. In the acute phase (Figure 5a), the AO group only inhibited protein oxidation by 18.11% ± 9.91; meanwhile, CUR conferred protection to proteins by inhibiting oxidative damage by 99.59% ± 0.168 (*p* < 0.0001) in the AP group. Similar protection was observed in the AT group (97.17% ± 0.137). When the chronic phase was complete (Figure 5b), the CO group showed antioxidant protection of 18.17% ± 4.87. In contrast, dietary CUR protected against oxidative protein damage in the CP, with a percentage of 99.50 ± 0.038 (*p* < 0.0001), and for the CT group, the percentage was 96.48 ± 0.34 (*p* < 0.0001). The percentage of protein oxidative damage in the CI group was 95.91 ± 1.83, and for the CC, group the percentage was 98.82 ± 0.08.

## 3. Discussion

In this work, the preventive and therapeutic dietary administration of CUR was analyzed, as it exerted differentiated modulatory effects on the activity of the plasma antioxidant enzymes in rats during acute and chronic exposure to O_3_. However, the of CAT, SOD, and GPx activity was increased by CUR, with slight differences between preventive and therapeutic administration. Some of the differences that were identified among the measured enzymes may respond to dynamic regulations within the antioxidant system depending on whether or not it is under the influence of curcumin. Contrarily, CUR inhibited the oxidative damage caused by O_3_, regardless of the exposure time or the CUR administration mode. In this case, the direct antioxidant activity of CUR as a scavenger for RONS should be highlighted.

The variability found in diverse reports analyzing the effects of CUR or its derivatives with respect to the behavior of endogenous antioxidant enzymes may be due to factors such as administration mode (preventive, therapeutic, continue, intermittent, or single dose), dose level (high, medium, or low), the age and gender of the animals used in experimental models, injury mode (single, continuous, intermittent, acute, chronic), the nature of the injury agent (physical, chemical, mechanic), and the experimental design, among other factors [29,30,31,32,33,34].

In our experiments, we obtained dramatic results for the CAT, SOD, and GPx activity. Some differences are important to clarify with respect to the age of the animals and the activation sequence. The basal activity of CAT remained unaltered in the acute versus the chronic phase in the intact group as well as in the CUR-fed group. Contrarily, the group exposed to O_3_ exhibited a significant decrease in CAT activity during both exposure phases (49% acute phase, and 68% chronic phase). The SOD activity in the intact group during the acute phase was not different from the activity determined for the O_3_-exposed group; for its part, CUR improved SOD activity in the AC group. Furthermore, SOD activity was not affected by O_3_ during the acute phase, as it could had been protected by GPx, which showed normal activity in this exposure phase; concomitantly, the CAT activity was depleted at the same time, and this could indicate that it was consumed at this point. Similar behavior has been previously reported [3]. In contrast, the protective effect of CUR administered in the preventive or therapeutic mode induced a recovery of the CAT, SOD, and GPx activity at levels similar to those in the intact condition. These results are in accordance with previous reports [35].

As a plausible explanation, the decreased CAT, SOD, and GPx activity could be due to the low activation of Nrf2 [36] as consequence of increased activation of NFkB by RONS. Previous studies have documented that the activation of NFkB leads to an overexpression of KEAP1, which binds Nrf2, inhibiting its translocation and increasing its proteosomal degradation [9,37]. This could be a possible explanation for the decline in the activity of the antioxidant endogenous enzymes. In spite of this mechanism having been previously reported, it remains to be further documented in similar studies. Furthermore, RONS may cause oxidative damage to CAT at high levels, reducing its activity. In our experiments, the exposure to O_3_ caused a significant decrease in CAT activity in the AO and CO groups; meanwhile, the AI and AC groups showed high basal activity, similar to the preventive and therapeutic groups that recovered their CAT activity due to the effects of CUR. Furthermore, the SOD and GPx activity was not affected in the acute phase, which could be due to the predominant formation of H_2_O_2_ caused by O_3_ and its neutralization by CAT, whose activity was exhausted during acute exposure. On the contrary, the activity of both enzymes was decreased by O_3_ in the chronic phase. This effect could be the result of the activation of NFkB, leading to the increased expression of KEAP1, which, in turn, binds to Nrf2, leading to its further proteosomal degradation and the consequent decreased expression of CAT, SOD, and GPx [38].

In the preventive mode, CUR could first act as an antioxidant by neutralizing RONS and then be involved in priming the activation of Nrf2 through the reduction of serine residues, leading to its nuclear translocation [9]. Furthermore, CUR can avoid the activation of NFkB through the inhibition of the I kappa B kinase [39,40]; this would decrease NFkB dissociation from IkBa and would consequently also decrease the expression of KEAP1, leading to reduced Nrf2 proteosomal degradation [41]. Thus, when the oxidant insult by O_3_ begins, both transcriptional factors are modulated by CUR. In the therapeutic mode, CUR exerts action focused on reverting the activation of NFkB and inhibiting the degradation of Nrf2 by reducing the expression of KEAP1, favoring its activation and reactivating the expression and activity of CAT, SOD, and GPx [8,9].

The SOD activity in the O_3_ control group in the acute phase did not exhibit significant activity modification. This could mean that SOD activity may have been restored every day after the 4 h of daily exposure. However, after O_3_ exposure for 60 days (chronic phase), the SOD activity was no longer restored and caused a 50% decrease. Similar findings have been previously reported [4,42]. On the other hand, the intact control in the chronic phase presented increased SOD activity (64.47%) compared to the acute phase; this could indicate the maturation of the naturally occurring endogenous antioxidant system, as previously shown [43]. In contrast, the beneficial effect of CUR was evidenced in the therapeutic group in both phases by increasing the enzyme activity. These findings agree with previous reports [44,45]. Furthermore, this is the first time that the effect of CUR supporting the activity of SOD against the damage caused by acute or chronic exposure to ozone in a preventive mode has been reported.

In the acute phase (15 days), the GPx activity was not significantly altered by O_3_ exposure, which could be due to a repairing process during the 20 h interval without exposure. However, GPx activity was significantly increased when the animals were exposed to ozone and treated with CUR in the preventive and therapeutic approaches. Furthermore, the chronic exposure to O_3_ caused a significant decrease in GPx activity, meaning that the 60-day exposure to O_3_ impeded the success of the repair process. This decrease was reverted by CUR in the therapeutic mode and was avoided in the preventive mode.

The GPx activity in the chronic phase of the O_3_ control group showed a 50% decrease in activity in the chronic phase compared to in the acute phase, as described in vitro by [3].

Using this model of oxidative damage at an O_3_ dose of 0.7 ppm for four hours (h) every day, the CO group showed that it can increase oxidative damage in the cell membrane by increasing MDA, 4-HNE, and protein carbonylation [8,42] in such a way that it allows the over-activation of NF-κB, which induces the overexpression of KEAP1. In this context, when KEAP1 sequesters Nrf2 in such a way that it decreases the activity of antioxidant enzymes [9], this allows the onset of oxidative stress. When Nrf2 stops translocating to the nucleus, the activity of antioxidant enzymes decreases, as demonstrated in this work and as previously documented [3].

To assess the estimation of oxidative damage to lipids, MDA and 4HNE metabolites are commonly determined, and these are generated during RONS interaction with cells. The implemented methods report the use of mesylate to estimate both metabolites simultaneously [46]. The results demonstrating the oxidative damage to lipids obtained in the AP, CP, AT, and CT groups showed that CUR maintained similar percentages of oxidative inhibition similar to the intact group in both phases. These findings indicate that CUR performed its direct antioxidant effect as an RONS scavenger and indirectly mediated Nrf2 activation and NFκB inactivation. This effect is very similar to that reported in the work carried out by Mendoza-Magaña et al., 2021, and Guerrero-Hue et al., 2019 [27,47]. Furthermore, the results of the protein carbonylation assay also showed that CUR was able to inhibit this oxidative damage process in the experimental groups that were exposed to ozone and fed with CUR in the preventive and therapeutic modes. These findings have been reported [48,49].

Supported by the findings and analysis performed in this report and other studies carried out previously, we propose testing the protective effects that CUR may exert in human populations inhabiting in cities with continuously high ozone levels throughout the year and evaluating its effects on the endogenous antioxidant system, which would improve the inhibition of oxidative damage, cognitive performance, and academic profile in volunteers of different ages. Considering that CUR may prevent and revert oxidative damage due to its scavenger properties and because of the enhancement of the antioxidant enzymes activity evaluated in the present work, its beneficial effects should continue to be evaluated in experimental models and clinical trials. We suggest that it might be used to prevent pollutant-related diseases, such as Parkinson’s and Alzheimer’s disease, as well as a complimentary therapy for these oxidative damage-related disorders.

## 4. Materials and Methods

### 4.1. Animals

This study was performed using 50 male Wistar rats (*Rattus norvegicus*) that were 21-days-old and that had an average body weight of 130 g. They were kept in light and dark cycles of 12 X 12 h and at 50–60% relative humidity with free access to food and water. The experiments were carried out according to the National Institutes of Health Guide for the Care and Use of Laboratory Animals (NIH Publications No. 8023, revised 1978), which are as well established by the Ethics Committee of the Health Sciences Center (CUCS, Universidad de Guadalajara), under the approval number, CI-00512, and by the Ministry of Health of Jalisco State 76/UG-JAL/2011.

### 4.2. Diet

A turmeric ethanolic extract was obtained using a Soxhlet extraction apparatus. The infrared spectrum profile was compared to a CUR standard diluted in ethanol (Sigma Aldrich, St. Louis, MO, USA), showing and confirming the identity of the extracted molecule (Figure 1). The concentration of CUR in the extract was determined by UV spectrometry and adjusted to 12 mg/mL. This solution was employed to impregnate the food pellets (Prolab^®^RMH Laboratory Animal diet, 2500 Rodent 5P14), and ethanol was eliminated by ventilated evaporation at 58 °C for 4 h. These procedures were performed in the dark to avoid CUR photodegradation. This food provided an approximate daily CUR dose of 5.6 mg/Kg. The diet was dynamically adjusted by increasing the grams of food served according to the increases in the animal´s body weight.

### 4.3. Experimental Design

Animals were randomly distributed into five experimental groups of 10 rats each. Every experimental group was adapted to the handling procedures and accommodation in the exposure acrylic chamber for four hours over a 7-day period prior to initializing the experiment. The adaptation was carried out to minimize the effect of human contact. The design was established by considering two periods of O_3_ exposure: an acute phase (A, 15 days) and a chronic phase (C, 60 days), which led to the formation of two subgroups. Thus, the final experimental groups were named as follows: acute intact group (AI, *n* = 5) and chronic intact group (CI, *n* = 5) were exposed to O_3_-free air and food without CUR; the groups fed with CUR supplementation and exposed to O_3_-free air in both phases were denoted as AC (*n* = 5) and CC (*n* = 5). The O_3_-exposed groups were exposed to 0.7 ppm for 4 h and were denoted as AO (*n* = 5) and CO (*n* = 5). Time of exposure to O_3_ and/or the time of the supplemented diet with CUR defined the following groups as the therapeutic groups or the preventive groups for each exposure period. The preventive groups received food with CUR supplementation for the first 7 days, and afterwards, O_3_ exposure began and continued until both exposure phases were completed (AP, *n* = 5 and CP, *n* = 5). The therapeutic groups were exposed to 0.7 ppm of O_3_ for the first 7 days, and afterwards the diet with CUR supplementation was served until the end of each exposure time, defining the groups as therapeutic acute (AT, *n* = 5) and therapeutic chronic (CT, *n* = 5).

The Table 1 summarizes the experimental groups and their nomenclature.

### 4.4. Ozone Exposure

Experimental were groups exposed to O_3_ (0.7 ppm), as this dose was previously reported to cause antioxidant defense depletion [50], or to O_3_ free air and were placed inside a hermetic acrylic chamber (65 × 25 × 45 cm L/H/D) coupled to a premix chamber (40 × 24 × 45 cm) daily for 4 h; the exposure chamber was built using a similar design as previously reported [8,51,52]. The premix chamber received O_3_ generated by a Certizon C100 apparatus (Sander Elektroapparatebau GmbH, Uetze, Alemania), which was fed with medical-grade oxygen. The O_3_ generated was mixed with O_3_-free air to adjust the flux to the mentioned concentration. The concentration of O_3_ was monitored with a semiconductor (ES-600, Ozone Solutions Inc., Hull, ID, USA) to yield an adequate atmosphere with constant flow of 1.6 to 1.2 L/min. As part of biosecurity actions, the released O_3_ from the chamber was inactivated with neutralizing filters made of a sodium nitrate, potassium carbonate, glycerol, methanol, and water solution before being released into the air.

### 4.5. Plasma Sample Obtention

Once the experimental groups concluded their exposure phase, the animals were euthanasia via intraperitoneal injection with a lethal dose of sodium pentobarbital (90 mg/Kg). Blood was extracted via intracardiac punction using heparinized syringes; plasma samples were separated by centrifugation at 2500 rpm at 4 °C, and 10 µL/mL of phosphate-buffered solution containing a homemade antiprotease cocktail (bestatin, leupentin, aprotinin, PMSF, EDTA and EGTA) was added to each sample (Sigma Chemical, St. Louis, MO, USA). Samples were frozen at −80 °C and stored until use.

### 4.6. Hippocampus Processing

Immediately after blood collection, the animals were decapitated, and the head was placed on an ice-cold surface while the hippocampus was dissected. The hippocampus has been reported to be a brain structure that is highly sensitive to oxidative damage at a dose of 0.7 ppm of O_3_ [50]. Two hippocampi were weighted separately to prepare two 10% homogenate samples in PBS. A 10 µL/mL amount of 0.5 M butylhydroxytoluene (BTH) was added to samples used for malondialdehyde and 4 hydroxynonenal (MDA/4-HNE) determination. The other received 10 µL/mL of an antiprotease cocktail with 0.2 mM mercaptoethanol (Sigma Chemical, St. Louis, MO, USA) for protein carbonylation analysis. Tissue samples were frozen at −80 °C and stored until use [8].

### 4.7. Estimation of Antioxidant Enzyme Activity

The SOD, CAT, GPX activity in plasma samples from the experimental groups obtained after completing the acute and chronic exposure times was determined using a commercial kit according to the manufacturer’s instructions (Abcam, Cambridge, UK). Plasma samples were stored at −80 °C until analysis; subsequently, the homogeneity of the protein content was determined by the Lowry method. This assay is based on the Biuret reaction and has additional steps to increase the sensibility. In the biuret reaction, copper interacts with four nitrogen atoms of the peptides to form a cuprous complex. The Lowry method adds phosphomolybdic/phosphotungstic acid, which is also known as the Folin–Ciocalteu reagent. This reagent interacts with cuprous ions and the side chains of tyrosine, tryptophan, and cysteine to produce a blue-green color that can be detected between 650 and 750 nm [52]. Once analyzed, all samples are diluted to the same concentration as before the enzyme activity assays to avoid variation due to different protein contents.

The analysis of CAT activity is based on a two-step procedure. The samples containing CAT were incubated in the presence of a known concentration of H_2_O_2_; the unconverted H_2_O_2_ reacts with the OxiRed probe, producing a product measurable at 570 nm. Therefore, the catalase activity contained in the sample is reversely proportional to the optical density obtained. The procedure was performed according to the kit’s manufacturer’s instructions (ab83464).

The SOD activity is measurable through the superoxide anions produced by xanthine oxidase activity. Superoxide anions are dismutated by SOD, generating hydrogen peroxide and oxygen. Superoxide anions act on the tetrazolium salt WST-1 to produce formazan dye, which is soluble in water and measurable at 450 nm. Thus, greater SOD activity is revealed in the sample by the lower amount of the formazan dye produced. The procedure was carried out according to the manufacturer’s instructions. Furthermore, the results are expressed as the activation percentage rate, as indicated by the manufacturer (ab65354).

The GPx activity is based on the reduction of cumene hydroperoxide while it oxidizes glutathione (GSH) to glutathione disulfide (GSSH). GSSH is reduced to GSH due to the consumption of NADPH by glutathione reductase (GR). Thus, the decrease in NADPH at 340 nm is proportional to the GPx activity. The procedure was performed according to manufacturer’s instructions (ab102530).

All plasma samples were tested in duplicate for the mentioned assays. Determinations were performed in a microplate reader (Multiskan Go., Thermo Scientific, Waltham, MA, USA).

### 4.8. Inhibition of MDA and 4-HNE Formation by CUR

The method to determine the concentration of oxidized lipid metabolites in hippocampus homogenate samples was carried out according to manufacturer´s instructions (Cat. # FR12, Oxford Biomedical Res., Oxford, MI, USA). In brief, samples were centrifuged at 3000× *g* for five min at 4 °C, and 250 µL of the sample was added in each centrifuged tube. Afterwards, 812.5 µL of 11-Methyl-2-phenylindole was added and mixed and incubated at 45 °C for 40 min. Subsequently, 187.5 µL of mesylate was added and quenched in an ice bath. The samples were incubated at 45 °C over 45 min. Finally, the samples were maintained at 4 °C, and 200 µL of the supernatant was placed in a 96-well microtiter plate reader and analyzed at λ 595 nm absorbance in triplicate. The calibration curve was prepared using 650 µL of chromogen solution with progressive concentrations with malonaldehyde bis (dimethyl acetal) at a concentration of 0.315 and with 10 nmol/mL. To assess the estimation of oxidative damage to lipids, MDA and 4HNE metabolites are commonly determined, and these are generated during RONS interaction with cells. The implemented methods report the use of mesylate to estimate both metabolites simultaneously [46]. Results are expressed as the percentage inhibition of oxidative damage by considering a 100% the level obtained in the AO and CO groups.

### 4.9. Protein Carbonylation

The protein carbonylation assay reveals the level of oxidative damage to proteins induced by O_3_ exposure. The OxyBlot kit was used according to manufacturer’s instructions (Cat. # S7150, Merck Millipore Corp., Billerica, MA, USA, EE. UU.).

The protein concentration in the samples was determined by the Bradford method, and 4 µg of protein/µL was used. Duplicate samples were denaturalized with 5 µL of 12% sodium lauryl sulphate added to each sample. Half samples from each group were incubated with the derivatizing reagent 2,4-Dinitrophenylhydrazine (DNPH). The remaining samples were incubated with a non-derivatizing control reagent. Afterwards, the reaction was halted, and samples were electrophoresed in 10% of SDS–polyacrylamide gels using a mini-PROTEAN chamber (Bio-Rad, Hercules, CA, USA) at 100 V. Consecutively, proteins were electrotransferred to PVDF membranes using a blot module (Bio-Rad, Hercules, CA, USA) at 25 V for 12 h at 4 °C. At the end, the PVDF membranes with the transferred proteins were blocked with 5% of skimmed milk in PBS for 12 h at 4 °C. Following these steps, samples were incubated with rabbit IgG anti-DNPH (1:150). Peroxidase labelled IgG anti-rabbit (1:500) was used as a secondary antibody, and incubation was performed for 1 h at room temperature. After washing, the membranes were exposed to HRP quimioluminiscent Immobilon reagent (Millipore Corp., Billerica, MA, USA, EE. UU.) to visualize the oxidized protein bands. Analysis of the blot images was performed using Image Studio Lite Ver 5.2^®^, and the integrated optical density (IOD) data were generated per sample and per experimental group to perform the statistical analysis. The results were expressed in the percentage of inhibition of oxidative damage to proteins.

## 5. Conclusions

The dietary use of CUR showed high efficiency as a regulator of antioxidant enzymes and a protector of the oxidative damage caused by O_3_. These findings have started to show that CUR can be useful against the environmental contingencies that usually occur in cities with high atmospheric pollution.

## Figures and Tables

**Table 1:** Experimental groups and design.

	Acute Phase
AO	Acute O_3_ (exposure to 0.7 ppm of O_3_ for 4 h for 15 days).
AI	Acute intact (exposed to O_3_ free air for 4 h and without CUR supplementation for 15 days).
AC	Acute CUR (diet supplemented with CUR and exposed to O_3-_free air for 15 days).
AP	Acute preventive (diet supplemented with CUR provided 7 days prior to exposure to O_3_ for 15 days and continued CUR supplementation).
AT	Acute therapeutic (exposure to O_3_ 7 days prior to the administration of the diet supplemented with CUR for 15 days and continued exposure to O_3_ until day 15).
	Chronic phase
CO	Chronic O_3_ (exposure to 0.7 ppm of O_3_ for 4 h for 60 days).
CI	Chronic intact (exposure to O_3_-free air for 4 h and without CUR supplementation for 60 days).
CC	Chronic CUR (exposure to O_3_-free air for 4 h with diet supplemented with CUR administered for 60 days).
CP	Chronic preventive (diet supplemented with CUR provided 7 days prior to exposure to O_3_ for 60 days; CUR-supplemented feeding continued until day 60).
CT	Chronic therapeutic (exposure to O_3_ for 7 days prior to the administration of the diet supplemented with CUR for 60 days; exposure to O_3_ continued until day 60).

## Data Availability

Data supporting reported results can be found in the Research Coordination Department of the Centro Universitario de Ciencias de la Salud and with the Corresponding Author.

## Sample Availability

Samples of the compounds are not available.
